# Risk Factors and Preventive Strategies for Alzheimer’s disease and Related Dementias

**DOI:** 10.1093/geroni/igaf122.2173

**Published:** 2025-12-31

## Abstract

This session provides insights into trajectories of risk factors for dementia as well as potential targets for Alzheimer’s and Related Dementia prevention.

